# B7-H3 and B7-H1 expression in cerebral spinal fluid and tumor tissue correlates with the malignancy grade of glioma patients

**DOI:** 10.3892/ol.2014.2268

**Published:** 2014-06-19

**Authors:** APARAJITA BARAL, HONG XING YE, PU CHA JIANG, YU YAO, YING MAO

**Affiliations:** 1 Department of Neurosurgery, Zhongnan Hospital, Wuhan University, Wuhan, Hubei 430071, P.R. China; 2 Department of Neurosurgery, Huashan Hospital, Fudan University, Shanghai 200040, P.R. China; 3 Institute of Biomedical Sciences, Fudan University, State Key Laboratory of Medical Neurobiology, Shanghai 200032, P.R. China

**Keywords:** glioma, B7 family, B7-H3, B7-H1, cerebral spinal fluid, blood serum

## Abstract

The B7 family consists of activating and inhibitory molecules that regulate immune responses. Recent research demonstrated the roles of soluble B7-H3 (sB7-H3) and soluble B7-H1 (sB7-H1) in the blood serum of various tumors; however, none of these studies investigated the expression of these proteins in the cerebral spinal fluid (CSF) and blood serum of patients with glioma. The aim of the present study was to investigate the expression of B7-H3 and B7-H1 in the CSF, blood serum and tissues of patients with glioma and their correlation with clinicopathological data. Between January 2012 and November 2012, samples were obtained from 78 patients with glioma, four CSF samples were obtained from patients with a moderate traumatic brain injury, four brain tissue samples were obtained from patients with a traumatic brain injury and 40 blood serum samples were obtained from healthy individuals. The expression of B7-H3 and B7-H1 in the CSF, blood serum and tumor samples of the patients with high-grade glioma was found to be higher than that in the patients with low-grade glioma. However, no significant differences in sB7-H3 and sB7-H1 expression were observed in the blood serum of the patients with glioma compared with the healthy control subjects. In addition, the expression of sB7-H3 and sB7-H1 in the CSF of the patients with glioma was higher than that in the CSF of the patients with a moderate traumatic brain injury. Furthermore, in the patients with glioma, B7-H3 and B7-H1 expression in the CSF and tumor tissue, although not in the blood serum, correlated with the glioma grade.

## Introduction

Due to the poor survival rate of patients with glioma, particularly those with high-grade glioma (HGG), a number of studies have investigated the different groups of the B7 family (1). The B7 family belongs to the immunoglobulin superfamily, which consists of B7-1, -2, -H1, -H2, -DC, -H3 and -H4 (2). The B7 family has important implications for the treatment of cancer, transplantation and autoimmune diseases (3). B7-H3 is reported to be released by monocytes, dendritic cells and activated T cells (4). It is a tumor-associated antigen, which regulates important cellular responses, including proliferation, apoptosis, adhesion, tumor metastasis and immunity (5–9), thus demonstrating its novel biological role in tumor progression and metastasis (9). B7-H3 protein expression has been detected in various types of tumor, including ovarian, lung, stomach, prostate and pancreatic tumors, as well as clear cell renal and colorectal carcinoma (10–15). Lemke *et al* (16) reported that B7-H3 expression is present in human glioma tissues (16). Although its function remains uncertain, its expression has been associated with disease progression. B7-H1 is an important regulator of antitumor immunity (2). B7-H1 is expressed in hematopoietic malignancies, including leukemia, thymic neoplasms and multiple myeloma, as well as in the majority of types of solid human cancer, including breast, colon, esophageal, gastric, head and neck squamous cell, kidney, liver, lung, ovarian, pancreatic, salivary gland and urothelial carcinomas, as well as glioblastoma (tumor tissues only), Wilms’ tumor and melanoma (17–22). Furthermore, soluble B7-H1 (sB7-H1) expression has been detected in the blood serum of patients with renal cell carcinoma (23).

B7-H3 and B7-H1 are significant in the interaction between tumors and the immune system. This has been observed in numerous different tumor types (1,2,5,10–15,25,26), including glioma (16,27). The expression of sB7-H3 and sB7-H1 has previously been analyzed in the CSF and blood serum of patients with various types of tumor, however, not in those with glioma (10,23,24).

Sun *et al* (5), Chen *et al* (28) and Frigola *et al* (23) reported the expression of sB7-H3 and sB7-H1 in the blood serum of patients with colorectal carcinoma, the CSF and plasma of patients with bacterial meningitis and the blood serum of patients with renal cell carcinoma, respectively. However, these studies did not investigate the expression of sB7-H3 and sB7-H1 in the CSF and blood serum of patients with glioma. The aim of the present study was to evaluate whether patients with glioma have increased levels of B7-H3 and B7-H1 in their CSF, tumor tissue and blood serum and whether this correlates with the glioma grading.

## Patients and methods

### Patients and sample collection

A total of 78 patients (40 males and 38 females) with a median age of 45 years (age range, 18–70 years) who had been diagnosed with brain glioma and treated surgically at the Department of Neurosurgery, Huashan Hospital, Fudan University (Shanghai, China), between January 2012 and August 2012 were selected for inclusion in the present study, subsequent to obtaining Institutional Review Board approval. Patients provided written informed consent. The study was approved by the ethics committee of the Department of Neurosurgery, Huashan Hospital of Fudan University.

The preoperative collection of CSF and blood serum from the consenting patients was initiated at the Huashan Hospital of Fudan University in January 2012. CSF was obtained via lumbar puncture, and arterial and venous blood samples were obtained preoperatively in the operating theater of Huashan Hospital of Fudan University. Patients, who exhibited symptoms that indicated increasing intracranial pressure, including a severe headache, nausea, vomiting and papilloedema, were excluded due to the high risk associated with performing a CSF aspiration in these patients. Patients with a history of meningitis or a particularly large tumor were also excluded. Each blood sample was centrifuged (Sigma 3K30; Sigma-Aldrich Chemie Gmbh, Munich, Germany) at 20°C at 13,400 × g for 15 min and the blood serum was isolated. CSF and blood serum samples were stored at −80°C for the detection of B7-H3 and B7-H1 using enzyme-linked immunosorbent assay (ELISA); the patient health data and pathological features were undisclosed. CSF from four patients with a moderate traumatic brain injury [Glasgow Coma Scale (GCS) score, 9–12], as well as 40 healthy donors with no evidence of a history of brain tumor, served as controls and were analyzed. Tumor samples were collected from patients at the time of surgical resection at Huashan Hospital of Fudan University. Four brain tissue samples from patients with a moderate traumatic brain injury (controls) were obtained from the operating theatre at the Department of Neurosurgery at the Huashan Hospital of Fudan University. Samples were snap frozen in liquid nitrogen and stored at −80°C until use. Among the 78 cases, 55 CSF samples, 60 tumor samples and 78 blood serum samples were obtained for B7-H3 analysis, while 40 CSF samples, 43 tumor samples and 60 blood serum samples were collected for B7-H1 analysis (Table I).

### Quantitative sandwich ELISA of the CSF and blood serum

CSF and blood serum samples were allowed to clot for 30 min, prior to centrifugation (Sigma 3K30; Sigma-Aldrich Chemie Gmbh) for 15 min at 13,400 × g. The samples were aliquoted and stored at ≤20°C. Standards were reconstituted with 1 ml deionized water. For the investigation of B7-H3 (catalog no. DB7H30; R&D Systems, Minneapolis, MN, USA), 900 μl calibrator diluent RD6–41 was pipetted into 50 ng/ml tubes and 500 μl calibrator diluent RD6–41 was pipetted into the remaining tubes, and a dilution series was generated as follows: 50, 25, 12.5, 6.25, 3.12, 1.56, 0.781 and 0.00 ng/ml, which served as a standard. A total of 100 μl assay diluent RD1–109 was added to each well, and 50 μl B7-H3 standard plus the samples was added to each well. For the investigation of B7-H1 (catalog no. E0513; Wuhan EIAab Science Co., Ltd., Wuhan, China), 900 μl sample diluent was pipetted into 10 ng/ml tubes and 500 μl sample diluent was pipetted into the remaining tubes and a dilution series was generated as follows: 10, 5, 2.50, 1.25, 0.62, 0.31, 0.15 and 0.00 ng/ml, which served as a standard. A total of 100 μl B7-H1 standard plus the samples was added to each well. After a 2-h incubation period at room temperature on a horizontal orbital microplate shaker (0.12 orbit; mini size microplate shaker-2AD, Maple Lab Scientific, Dalang, China) set at 500 ± rpm, the plates were washed three times using wash buffer. A total of 200 μl B7-H3 or B7-H1 conjugate was added and incubated for 2 h at room temperature on the shaker. The plates were washed three times using wash buffer, decanted, inverted and blotted using clean paper towels. Subsequently, 200 μl substrate solution was added to each well and incubated for 30 min at room temperature in the dark. Stop solution [50 μl; B7-H3 (cat no. DB7H30; R&D Systems); B7-H1 (cat no. E0513; Wuhan EIAab Science Co., Ltd.)] was added to each well and the plate was gently tapped to ensure thorough mixing. The plates were read at an absorbance of 405 and 570 nm using a SpectraMax^®^ M2 fluorescence absorbance cuvette (Molecular Devices, LLC., Sunnyvale, CA, USA). The experiments were performed in duplicate and obtained similar results.

### Reverse transcription-quantitative polymerase chain reaction (RT-qPCR) analysis of brain tumor samples

For tissue preparation for the RNA and protein extraction, the tissue was homogenized using a pestle and mortar in liquid nitrogen. Total RNA was extracted using an RNA purification system (Qiagen, Valencia, CA, USA) and treated with RNase-free DNase I (Roche, Mannheim, Germany) to remove any genomic DNA. The complementary DNA was prepared from 5–6 mg total RNA using SuperScript^®^ RNase H-Reverse Transcriptase (Invitrogen Life Technologies, Carlsbad, CA, USA) and random hexamers (Sigma-Aldrich, St. Louis, MO, USA). For the qPCR analysis, gene expression was measured using an ABI Prism^®^ 7500 sequence detection system (Applied Biosystems, Foster City, CA, USA) with SYBR^®^ Green Master Mix (Eurogentec, Seraing, Belgium) and primers at optimized concentrations. The primers (Sigma-Aldrich) were selected to span exon-exon junctions. The sequences for human B7-H3 and human B7-H1 were as follows: Forward, 5′-CCTGCTGCCTTATTATTTCACA-3′ and reverse, 5′-CACTGCAAGAAGAGGGTGGT-3′ for 4IgB7-H3; and forward, 5′-GGACAAGCAGTGACCATCAAG-3′ and reverse, 5′-CCCAGAATTACCAAGTGAGTCCT-3′ for h-B7-H1. The DNA was amplified under the following conditions: 95°C for 1 min, followed by 35 cycles of 95°C for 30 sec and 72°C for 1 min. All results were normalized using glyceraldehyde 3-phosphate dehydrogenase (GAPDH). The primer sequences for the housekeeping gene, GAPDH were as follows: Forward, 5′-AGAAGGCTGGGGCTCATTTG-3′ and reverse, 5′-AGGGGCCATCCACAGTCTTC-3′. The PCR products were separated on a 1.2% agarose gel and visualized using ethidium bromide staining. Standard curves were generated for each gene and the amplification was 90–100% efficient. Relative quantification of gene expression was determined via comparison of the threshold and raw quantitative (RQ) values (the average RQ value) of each tumor sample. The experiments were performed in triplicate and obtained similar results.

### Immunohistochemistry (IHC)

Tumor tissues were collected at the time of surgical resection from the Huashan Hospital of Fudan University and were fixed using 4% paraformaldehyde. The tumor tissues were subsequently incubated in blocking buffer (2% horse serum, 0.2% Triton X-100 and 0.1% bovine serum albumin in phosphate-buffered saline; Sigma-Aldrich) for 1 h at room temperature. Sections were cut to 3 mm and were processed using a Ventana BenchMark XT immunostainer (Ventana Medical Systems, Inc., Tucson, AZ, USA). The staining procedure included a pre-treatment with Cell Conditioner 1 (pH 8; Sigma-Aldrich) for 60 min. For the detection of B7-H3, sections were incubated with goat anti-human B7-H3 antibodies (dilution, 1:200; R&D Systems) at 37°C for 32 min followed by rabbit anti-goat immunoglobulins (P0446; Dako, Glostrup, Denmark) for 32 min at room temperature. For the detection of B7-H1, sections were incubated with anti-B7-H1 antibodies (dilution, 1:200; MIH1; ebioscience Inc., San Diego, CA, USA) overnight at 4°C followed by incubation with fluorescein isothiocyanate-conjugated goat anti-mouse (dilution, 1:50; Kirkegaard & Perry Lab Inc., Gaithersburg, MD, USA) or rhodamine-conjugated goat anti-mouse (dilution, 1:150, Kirkegaard & Perry Lab Inc.) antibodies for 60 min. Nuclei were counterstained using 4′,6-diamidino-2-phenylindole. Incubation was followed by Ventana standard signal amplification, UltraWash to remove excess antibody, counterstaining with one drop of hematoxylin for 4 min and one drop of bluing reagent (Shandon Inc., Swickley, PA, USA) for 4 min. For visualization, an ultraView Universal DAB Detection kit (Ventana Medical Systems, Inc.) was used.

### Statistical analysis

Experiments were performed in duplicate or triplicate. Results are presented as the mean ± standard deviation and the χ^2^ test was conducted. Non-parametric tests, including the Kolmogorov-Smirnov and Shapiro-Wilk tests were also performed to compare data between the different groups. P<0.05 was considered to indicate a statistically significant difference. All statistical calculations were performed using SPSS for Windows (version 13. 0; SPSS Inc., Chicago, IL, USA).

## Results

### B7-H3 and B7-H1 expression in the CSF and blood serum

**Figure 1:**
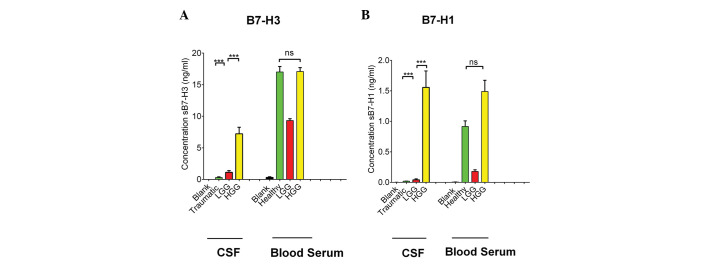
Quantitative sandwich enzyme-linked immunosorbent assay of the CSF and blood serum of patients with glioma. (A) Concentration of sB7-H3 and (B) sB7-H1 in the CSF and blood serum of patients with LGG, HGG and the control subjects. sB7-H3/H1, soluble B7-H3/H1; CSF, cerebral spinal fluid; LGG, low-grade glioma; HGG, high-grade glioma; ns, not significant. ^***^P<0.001.

ELISA revealed that the concentrations of sB7-H3 and sB7-H1 in the CSF were significantly lower in the patients with low-grade glioma (LGG; B7-H3, 1.129±1.256 ng/ml and B7-H1, 0.099±0.133 ng/ml) compared with the concentrations in patients with HGG (B7-H3, 7.228±6.063 ng/ml and B7-H1, 1.557±1.200 ng/ml). The CSF from four patients with moderate traumatic brain injury (GCS score, 9–12) served as a control and demonstrated that the levels of sB7-H3 and sB7-H1 were 0.306±0.218 and 0.019±0.003 ng/ml, respectively (Fig. 1A and B and Table II). This shows that sB7-H3 and sB7-H1 are expressed at greater levels in the CSF of the patients with glioma compared with the patients with a traumatic brain injury. The expression of sB7-H3 in the blood serum was 9.323±1.569 ng/ml in the patients with LGG, 17.090±4.278 ng/ml in the patients with HGG and 17.020±5.466 ng/ml in the healthy control subjects. Furthermore, the expression of sB7-H1 in the blood serum was 0.003±0.003 ng/ml in the patients with LGG, 1.489±1.008 ng/ml in the patients with HGG and 0.916±0.575 ng/ml in the healthy control subjects (Fig. 1A and B and Table II). These findings demonstrate that sB7-H3 and sB7-H1 were expressed at greater levels in the blood serum of patients with HGG compared with the patients with LGG, however, there was no significant difference identified in the expression of sB7-H3 and sB7-H1 in the blood serum of the patients with glioma compared with that of the healthy control subjects. This indicates that the expression of sB7-H3 and sB7-H1 may be significant in the CSF, but not in the blood serum in patients with glioma.

### B7-H3 and B7-H1 expression in glioma tissue

**Figure 2:**
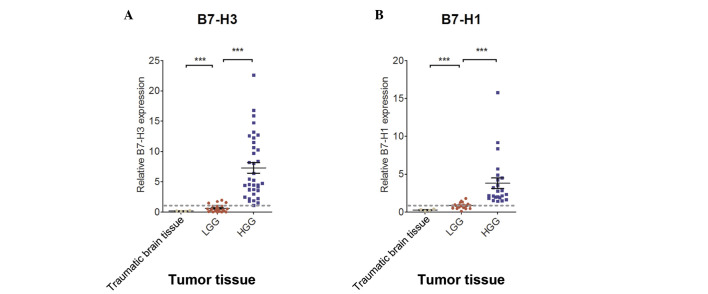
Polymerase chain reaction analysis of brain tumor samples in patients with brain glioma. RQ values of (A) B7-H3 and (B) B7-H1 expression in tumor tissue and traumatic brain injury tissue (control). The gray dotted line represents the control. LGG, low-grade glioma; HGG, high-grade glioma; RQ, raw quantitative. ^***^P<0.001.

RT-qPCR analysis revealed that the mean RQ values of B7-H3 and B7-H1 in the glioma tissue of the patients with LGG were 0.610±0.583 and 0.849±0.397, respectively, and in the patients with HGG were 7.287±5.207 and 3.813±3.350, respectively (Fig. 2A and B and Table II). The expression of B7-H3 and B7-H1 in the glioma tissue was found to be significantly correlated with LGG and HGG (P<0.001). Furthermore, the average RQ values of B7-H3 and B7-H1 in the moderate traumatic brain injury tissues were 0.173±0.064 and 0.273±0.116, respectively. These findings show that B7-H3 and B7-H1 were expressed at greater levels in the brain glioma tissue compared with the traumatic brain injury tissue.

### B7-H3 and B7-H1 expression is correlated with the glioma grade

**Figure 3:**
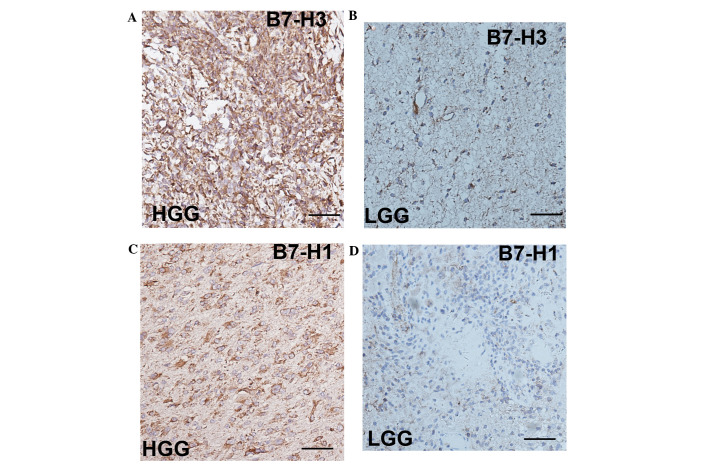
Immunohistochemistry of human glioma tissue (stain, hematoxylin and eosin; magnification, ×200). B7-H3 protein expression is higher in (A) HGG compared with (B) LGG. B7-H1 protein is higher in (C) HGG compared with (D) LGG. LGG, low-grade glioma; HGG, high-grade glioma.

IHC analysis revealed that the expression of B7-H3 and B7-H1 was found to be correlated with the glioma grade in freshly dissected human glioma tissue, which is consistent with the study by Lemke et al (16). The expression of B7-H3 and B7-H1 is demonstrated in Fig. 3 and was found to be increased in the HGG tissue (Fig. 3A and C) compared with the LGG tissue (Fig. 3B and D).

## Discussion

Previous studies have investigated B7-H3 and B7-H1 in different types of tumors (1,2,5,10–15,25,26), including in glioma tissues (16,27). Furthermore, recent studies have investigated the expression of B7-H3 and B7-H1 in the blood serum of patients with colorectal carcinoma and renal cell carcinoma (5,23), as well as in the CSF of patients with bacterial meningitis (28). However, to the best of our knowledge, no studies have investigated the expression of these proteins in the CSF and blood serum of patients with brain glioma. In the present study, significant sB7-H3 and sB7-H1 expression was observed in the CSF, although not in the blood serum, of patients with glioma. Table II shows the mean concentrations of sB7-H3 and sB7-H1 in CSF and blood serum samples and the RQ values of B7-H3 and B7-H1 expression in tumor tissue samples. In the present study, the expression of sB7-H3 and sB7-H1 was found to be higher in the CSF of patients with LGG compared with that in the patients with a moderate traumatic brain injury. In addition, the expression of sB7-H3 and sB7-H1 in the blood serum of the healthy individual was observed to be as high as that in the patients with glioma. Furthermore, in the brain tissues of the patients with a moderate brain injury, the expression of sB7-H3 and sB7-H1 was found to be lower than that in the brain tumor tissue of the patients with glioma and was also found to correlate with the glioma grade.

Sun *et al* (5) and Frigola *et al* (23) showed that sB7-H3 and sB7-H1 are expressed in the blood serum of patients with colorectal carcinoma and renal cell carcinoma. However, the present study identified no significant difference in the concentrations of sB7-H3 and sB7-H1 in the blood serum of the patients with brain glioma. This may be due to the different characteristic features and sizes of the proteins that pass through the blood-brain barrier and the receptor-mediated transcytosis to the central nervous system (24). This may explain why the expression of B7-H3 and B7-H1 may not be as significant in the blood serum of patients with brain glioma compared with that in patients with other tumors. In addition, with regard to the expression of B7-H3 and B7-H1 in the tumor tissue, the results of the present study were similar to those reported by Lemke *et al* (16) and our previous study (27), which showed that the expression of B7-H3 and B7-H1 in glioma tissue was correlated with the glioma grade. In the present study, a functional assay of the samples was not performed; however, such investigations will be performed in the future.

In conclusion, the present study demonstrates that in patients with glioma, the expression of B7-H3 and B7-H1 in CSF and tumor tissues, although not in blood serum, correlates with the glioma grade.

## Figures and Tables

**Table I:** Summary of the patient morphology from which the B7-H3 and B7-H1 samples were obtained.

	B7-H3 sample type			B7-H1 sample type		
		
Patient feature	CSF	Blood serum	Tumor tissue	CSF	Blood serum	Tumor tissue
Age (years)						
Median	44.5	45.0	44.5	44.0	45.0	44.0
Range	18–70	18–70	18–70	18–70	18–70	18–69
Gender (n)						
Male	33	40	36	22	34	20
Female	22	38	24	18	26	23
Total	55	78	60	40	60	43
Glioma (n)						
Classification						
LGG	21	30	24	20	30	20
HGG	34	48	36	20	30	23
Primary/Recurrent						
Primary glioma	51	74	56	36	56	39
Recurrent glioma and RN	4	4	4	4	4	4
Position of tumor (n)						
Frontal lobe	20	27	22	16	26	25
Parietal lobe	6	15	10	9	5	4
Temporal lobe	21	22	18	10	20	7
Occipital lobe	2	6	2	3	2	2
Other	6	8	8	2	7	5
Insular	1	2	2	1	2	2
Cerebellum	2	2	3	1	2	1
Putamen	2	2	2	0	1	1
Hippocampal	1	1	1	0	1	1
Thalamus	0	1	0	0	1	0

CSF, cerebral spinal fluid; LGG, low-grade glioma; HGG, high-grade glioma; RN, radiation necrosis.

**Table II:** B7-H3 and B7-H1 in the CSF, blood serum and tumor tissues of the cases and control subjects.

Biomarker	CSF (ng/ml)	Blood serum (ng/ml)	Tumor (RQ value)
B7-H3			
LGG	1.129±1.256	9.323±1.569	0.610±0.583
HGG	7.228±6.063	17.090±4.278	7.287±5.207
Control	0.306±0.218	17.020±5.466	0.173±0.064
B7-H1			
LGG	0.099±0.133	0. 003±0.003	0.849±0.397
HGG	1.557±1.200	1.489±1.008	3.813±3.350
Control	0.019±0.003	0.916±0.575	0.273±0.116

Data are presented as the mean ± standard deviation. CSF, cerebral spinal fluid; LGG, low-grade glioma; HGG, high-grade glioma; RQ, raw quantitative.

## Abbreviations

CSF: cerebral spinal fluid
LGG: low-grade glioma
HGG: high-grade glioma
PCR: polymerase chain reaction
ELISA: enzyme-linked immunosorbent assay
GAPDH: glyceraldehyde 3-phosphate dehydrogenase
IHC: immunohistochemistry
